# Analyses of Sox-B and Sox-E Family Genes in the Cephalopod *Sepia officinalis*: Revealing the Conserved and the Unusual

**DOI:** 10.1371/journal.pone.0157821

**Published:** 2016-06-22

**Authors:** Laura Focareta, Alison G. Cole

**Affiliations:** Biogem s.c.ar.l, Ariano Irpino, 83031, Italy; Laboratoire de Biologie du Développement de Villefranche-sur-Mer, FRANCE

## Abstract

Cephalopods provide an unprecedented opportunity for comparative studies of the developmental genetics of organ systems that are convergent with analogous vertebrate structures. The Sox-family of transcription factors is an important class of DNA-binding proteins that are known to be involved in many aspects of differentiation, but have been largely unstudied in lophotrochozoan systems. Using a degenerate primer strategy we have isolated coding sequence for three members of the Sox family of transcription factors from a cephalopod mollusk, the European cuttlefish *Sepia officinalis*: *Sof-SoxE*, *Sof-SoxB1*, and *Sof-SoxB2*. Analyses of their expression patterns during organogenesis reveals distinct spatial and temporal expression domains. *Sof-SoxB1* shows early ectodermal expression throughout the developing epithelium, which is gradually restricted to presumptive sensory epithelia. Expression within the nervous system appears by mid-embryogenesis. *Sof-SoxB2* expression is similar to *Sof-SoxB1* within the developing epithelia in early embryogenesis, however appears in largely non-overlapping expression domains within the central nervous system and is not expressed in the maturing sensory epithelium. In contrast, *Sof-SoxE* is expressed throughout the presumptive mesodermal territories at the onset of organogenesis. As development proceeds, *Sof-SoxE* expression is elevated throughout the developing peripheral circulatory system. This expression disappears as the circulatory system matures, but expression is maintained within undifferentiated connective tissues throughout the animal, and appears within the nervous system near the end of embryogenesis. SoxB proteins are widely known for their role in neural specification in numerous phylogenetic lineages. Our data suggests that *Sof-SoxB* genes play similar roles in cephalopods. In contrast, *Sof-SoxE* appears to be involved in the early stages of vasculogenesis of the cephalopod closed circulatory system, a novel role for a member of this gene family.

## Introduction

Invertebrate model systems are powerful tools for elucidating the genetic machinery underlying body patterning and cell type specification, in part due to the relative morphological simplicity of many invertebrate models (for example, the *Drosophila* larvae, *Caenorhabditis elegans*). However, invertebrate lineages such as cephalopod mollusks exhibit morphological and behavioural complexity that parallels vertebrates, thus providing the opportunity to study the developmental genetics of organ systems that are convergent with analogous vertebrate structures. For example, amongst a handful of cartilage-bearing invertebrate lineages, cephalopod mollusks possess cellular cartilages that are remarkably similar to vertebrate cartilages in terms of histology and developmental patterns Cole and Hall [1],[2],[3], have the most sophisticated centralized nervous system found outside vertebrate lineages (reviewed in [4]), and possess a sophisticated closed circulatory system that is unparalleled in other invertebrate lineages. However, knowledge of the molecular control of the development of any of these organ systems is only recently being brought to light ([5], [6] [7], [8]; [9]; [10]; [11], [12]; [13]). These types of data are crucial for the understanding of the evolution of morphological novelties, and to increase our understanding of how genetic circuitry can be recruited and expanded to independently give rise to convergent complex structures.

One group of genes that are known to play important roles in tissue specification and differentiation are members of the SOX family of transcription factors. The Sox-family genes are widely expressed throughout development and the different family groups show divergent functions in a broad range of tissue types. Here we describe for the first time the characterization of two of the Sox family groups in a cephalopod mollusk: SoxB and SoxE.

The SoxB family group can be further divided into two sub-groups: SoxB1 (Sox1, 2, and 3 in mammals) and SoxB2 (Sox14 and 21 in mammals). Within vertebrates, the SoxB1 genes are involved in control of dorsal-ventral patterning and gastrulation [14], and stem-cell maintenance (reviewed in [15]) in particular within the nervous system (reviewed in [16]). Amongst invertebrate lineages, expression data is limited. In amphioxus SoxB1 genes are expressed in the neural ectoderm as well as the fore and hind guts [17], as is the SoxB gene isolated from the planarian flatworm *Dugesia japonica* [18]. SoxB1 is expressed in ectodermal territories in embryos and larvae of the marine snail *Patella vulgata* [19], and during the early stages of embryogenesis in the coral *Acropora millepora* [20]. Foregut endoderm expresses SoxB1 in sea urchin embryos [21]. SoxB1 shows expression in the anterior pole of larvae of the cnidarian *Nematostella vectensis* [22] and in a horseshoe-shaped pattern that includes the region around the statocyst in juveniles of the acoelomate worm *Symsagittifera roscoffensis* [23]. SoxB2 genes also show conserved expression within neural ectoderm. The two vertebrate SoxB2 genes (Sox14 and Sox21) show specific, non-overlapping expression within specific areas of the developing nervous system (reviewed in [16]). Amongst invertebrates, genes of the SoxB2 class are expressed within the neuroectoderm in polychaetes, [24], arthropods: [25], bryozoans [26], hemichordates [27], and amphioxus [28].

The SoxE family genes (vertebrate Sox 8, 9, and 10) are less well studied amongst invertebrate lineages. Within vertebrates the SoxE genes (Sox8, Sox9, and Sox10) are expressed within mesodermal derivatives, including the kidney [29] and the somatic portion of the male gonads [30]. SoxE genes are involved in skeletogenesis, with Sox9 playing a key role in the activation of chondrocyte differentiation [31]. Sox9 is also involved in vertebrate endoderm formation, including the endodermally derived organs (lungs and pancreas, [32]). Vertebrate SoxE genes are also involved in the differentiation of the neural ectoderm, in particular the development of oligodendrocytes (reviewed in [16]). In addition, all three SoxE genes are all involved in neural crest specification [33], [34]. Within invertebrate lineages, SoxE in the hemichordate is expressed within the pharyngeal endoderm [35], whereas within the sea urchin SoxE expression is found within the mesodermal pouches that give rise to the adult body [36]. In *Drosophila*, the SoxE homolog (Sox100B) is expressed within the gut epithelium, the kidneys, and the somatic tissues of the gonad [37]. The SoxE ortholog is also expressed within the mesodermal blastema of a bryozoan [26], within early germ cells of the Pacific oyster [38], and is testes specific in the honeybee [25]. Interestingly, in cnidarian species SoxE genes are expressed in endodermal territories [20], [39]. We demonstrate here that SoxB and SoxE genes show expression at the onset of organogenesis in tissue restricted patterns, and show expression domains within the central nervous system in later embryogenesis.

## Materials and Methods

### Ethics Statement

Cephalopods are highly intelligent invertebrate animals and as such require special consideration [40] [41]. No permits were required for this work because the use of embryonic cephalopod material for research is currently not legislated in Europe. All embryos used in this study were lethally anesthetized in ethanol prior to sacrifice at stages prior to yolk absorption.

### Animals

Embryonic material used in this study come from two independent sources: fertilized eggs naturally deposited on fishing lines were obtained from the ISMAR-CNR in Ancona Italy; freshly deposited eggs from adult *Sepia officinalis* females maintained in aquaria were obtained from the Animal Services of the Stazione Zoologica ‘Anton Dohrn di Napoli (SZN). Fertilized eggs were kept in a closed salt water aquarium, as described in [5]. Egg capsules were opened in DEPC-treated artificial sea water (TETRA Marine SeaSalt), and isolated embryos were staged according to [3]. Three same-staged embryos were placed in TRIzol Reagent (Invitrogen, 15596–018) for each embryonic stage between stages E15 and E30, and stored at -80°C until used for RNA extractions. Embryos collected for whole mount *in situ* hybridization from stage E18 were pre-fixed overnight at 4°C in 4% PFA in DEPC-ASW within the innermost chorion layer, then manually dechorionated and post-fixed overnight in 4% PFA. Embryos from stages E24 to E28 were dechorionated and fixed overnight at 4°C. Fixed embryos were stored in 100% methanol at -20°C until use. Embryos collected for *in situ* hybridization in section were immediately embedded for sectioning as described in [5].

### Isolation of Sox family genes

mRNA of *S*. *officinalis* was extracted from staged embryos using TRIzol Reagent and cDNA was retro-transcribed with SuperScript III First-Strand Synthesis Super Kit (Invitrogen 18080–400), according to the manufacturer’s instructions. To identify SOX family members, a mix of cDNA derived from total RNA extractions, stages E24-E28, was amplified with degenerate primers from published literature that were designed to amplify the HMG domain of Sox proteins: forward: CCNATGAAYGCNTTYATG [35] and reverse: GGCTGRTAYTTRTAITCIGGRTRRTC [42]. Degenerate PCR products were amplified for 40 cycles (annealing temperature 42°C). The resultant 218 bp product was cloned into a pCRII-TOPO vector (Invitrogen, K4600-01), and a selection of positive colonies were sequenced. Gene identity was determined through identification of key family residues [43] and confirmed by phylogenetic analysis of aligned HMG domains using the online software: “CLUSTAL W2 phylogeny” with the UPGMA clustering method. (http://www.ebi.ac.uk/Tools/phylogeny/clustalw2_phylogeny/). We used two human TCF family sequences as outgroup in this analysis [43]

### Rapid Amplification of cDNA Ends

Coding sequences were extended towards the 3-prime end using the 5’/3’ RACE kit 2^nd^ Generation (Roche 03 353 621 001), using two rounds of gene specific amplification with the following primers: *SofSoxB1*: SoxB1_fw1: CTCGCGGACAAAGAAGAAAA, SoxB1_fw2: TGAAATCAGCAAACGTCTCG; *SofSoxE*: SoxE_fw1: CAGGCCGCTAGACGGAAG, SoxE_fw2: TGATCAATATCCCCATCTCCA; *SofSoxB2* SoxB2_fw1: CAGCGACGAAAAATGGCTCAGGAGA, SoxB2_fw2: TCGGCGCTGAATGGAAACTTCTCTC. Five-prime ends of the genes were amplified with a SMARTer™ RACE cDNA Amplification Kit (Clontech, 634924), using two rounds of gene specific amplification with the following primers: *SofSoxB1*: SoxB1_rv1: ACGGGAGATCTGAGGGAAAT, SoxB1_rv2: TTTCGCTTCGTCAATGAATG; *SofSoxE*: SoxE_rv2: CGTTGCTAACAGCTCCCTTC, SoxE_rv3: TATGTGGTTTCCGTTTCTCG; *SofSoxB2*: SoxB2_rv1: GAGGCCAGCGACGAAAAAT, SoxB2_rv1: GAGGCCAGCGACGAAAAAT. Amplified PCR products were separated on an agarose gel, specific bands were cloned into a pCRII-TOPO vector (Invitrogen, K4600-01) and sequenced to confirm clone identity and insert orientation.

### Reverse transcription polymerase chain reaction

RT-PCR amplification was performed according to [5] with the following primer pairs: *Sof-SoxB1* (SoxB1_rv3: ACACCGTCAGTCGTTGTAGC, SoxB1_fw3: ACGGGAGATCTGAGGGAAAT 122bp), *Sof-SoxE* (SoxE_fw3 TAGTCCCAACAACGGCTCTC, SoxE_rv4 AGCAAGATCGACTCGGCTAA 157bp), *Sof-SoxB2* (SoSoxB2_f3 AAATATGCATTTCCCCTGCCGGTCATT and SoxB2_r2 AGCCAGTGAACTGTGTGAGGT; 279bp) ribosomal protein S16 (RPS16 [44]: RPS16_fw GGTTTGACGAAGGTTTACCTG and RPS16_Rv CGCTGTTATCCCTATGGTAAC; 264bp).

### Quantitative polymerase chain reaction

qPCR amplifications were performed as described in [5] using the following gene specific primers: SoxB1_rv3 and SoxB1_fw3 as specified above (122bp); SoxE_fw4: CCTTTTATCGACGAGGCAGA and SoxE_rv5: CGTTGCTAACAGCTCCCTTC (109bp); SoxB2_f4: GGTTTCTTACCCCCAACGTC and SoxB2_r2 (as above; 156bp); RPS16_fw2 AAAAAGAAGTTTTAGTTGGGGTGA and RPS16_rv (as above; 158bp: endogenous control). Data were normalized to RPS16 levels, the expression level of which remains relatively constant in all the developmental stages examined [44]. Comparative gene expression levels of the normalized data were calculated as fold change relative to *Sof-SoxE* expression at stage E30, which showed the lowest levels of gene expression for all genes; standard errors in fold-change values were calculated with respect to standard deviations in the raw data according to [45].

### In situ hybridization

Digoxigenin (DIG)-labeled single-stranded RNA probes were synthesized from sequenced RACE product cDNA clones using the DIG RNA Labelling Kit (Roche 11 175 025 910) as recommended by the manufacturer: *Sof-SoxB1* (908bp– 3’RACE product); *Sof-SoxB2* (SoxB2_f3 and SoxB2_r3 as above; 494bp); *Sof-SoxE* (828bp– 3’RACE product). *in situ* hybridization on 10μm frozen serial sections followed the protocol described in [5]; control sections (sense probe and no-probe) were included in every assay.

Whole mount *in situ* hybridization (WMISH) was performed on a minimum of 3 same-staged embryos for each anti-sense probe used. Additional control embryos (sense probe and no-probe) were processed in parallel and included in every WMISH assay. Methanol-stored embryos were rehydrated in PBS, bleached with 3% H_2_O_2_ in 0.5% KOH for 1-12h according to the stage, post-fixed in 4% PFA–PBST for 1hr, incubated in PBST at 80°C for 15 minutes to destroy endogenous alkaline phosphatase activity, and placed in hybridization buffer (50% formamide, 2X standard saline citrate (SSC), 0.1% Tween 20 in DEPC treated water) without probes for 1-2h. Denatured RNA probes were then added at a concentration of 1ng/μl for each probe and incubated overnight at 65°C. Subsequently, embryos were subjected to a series of post-hybridization washes in decreasing concentrations of SSC with 0.1% Tween 20; incubated in blocking solution (Maleic acid pH7, 10mg/ml BSA, 0.1% Tween 20) for 1h, followed by incubation with anti-DIG-AP antibodies (Roche 11 093 274 910; 1:1500 in blocking solution) overnight at 4°C. Embryos were washed in PBST, equilibrated in alkaline phosphatase buffer (0.1M TRIS pH9.5, 0.1M NaCl, 0.1% Tween 20, 1mM Levamisole), and stained with NBT/BCIP (Roche 11 681 451 001) or BMPurple (Roche 11 442 074 001) until the colour reaction reached satisfactory intensity–control embryos were left in staining solution for the same time interval as those incubated with anti-sense probes. Embryos were passed through ascending concentrations of ethanol in PBS to remove background and darken the specific signal, re-hydrated in PBS, and viewed with a Carl Zeiss Stemi 2000C, photographed with CanonPower Shot A630.

## Results

### Cloning of Sox genes

**Fig 1:**
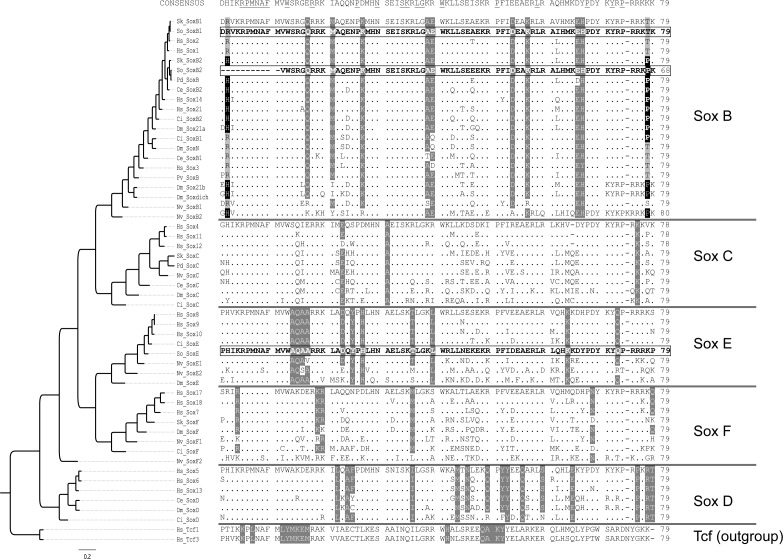
Identification of SOX family members through alignment and phylogenetic analysis of the conserved HMG domain. The phylogenetic tree on the left depicts the consensus relationships from analysis of the gene fragments (see methods). The alignment used for the analysis is shown on right; the consensus HMG sequence is shown above, with highly conserved residues underlined. Family specific amino acid positions are shaded grey. The first sequence from each family is presented in full, and only amino acids that differ from this sequence are shown in subsequent lines. The three isolated *Sepia officinalis* gene fragments are evidenced in bold typeface. The HMG domains of known SOX families fall out together in the tree as expected. Members of the SoxA family are known to be mammalian specific and are excluded from the analysis. We find no clear separation of the SoxB1 and B2 groups in our analysis, however residues specific to these two sub-families are present within the two isolated SoxB genes–SoxB2 residues are highlighted in black. Hs: *Homo sapiens*; Ce: *Ceanorhabditis elegans*; Pv: *Patella vulgata;* Sk: Saccoglossus kowalevskii; Dm: *Drosophila malangaster*; Ci: *Ciona instestinalis*; So: *Sepia officinalis*; Nv: *Nematostella;* Pd: *Platyneries dumerilii*; (“.”): conserved amino acid residues.

Degenerate primers designed against the conserved HMG domain were used to amplify and sequence a 218 bp fragment from cDNA derived from pooled embryonic RNA. Gene identity was determined by identification of key family-specific residues [43] and confirmed through phylogenetic analysis of the aligned HMG domain (Fig 1). All commonly recognized Sox family groups are supported in our analysis. The two SoxB-family genes are almost identical at the level of amino acid in the conserved HMG box, however differ at the nucleic acid sequence for 37% of the codons (20/53aa). Both *Sof-SoxB1* and *Sof-SoxB2* contain motifs identified by [46] that are specific to the SoxB1 and SoxB2 families respectively (Fig 1). *Sof-SoxB1* falls out in a clade that includes two of the three human orthologs (Sox1, and 2), whereas *Sof-SoxB2* is most similar to the *Platynereis* ortholog. *Sof-SoxE* is found nested within the SoxE clade.

**Fig 2:**
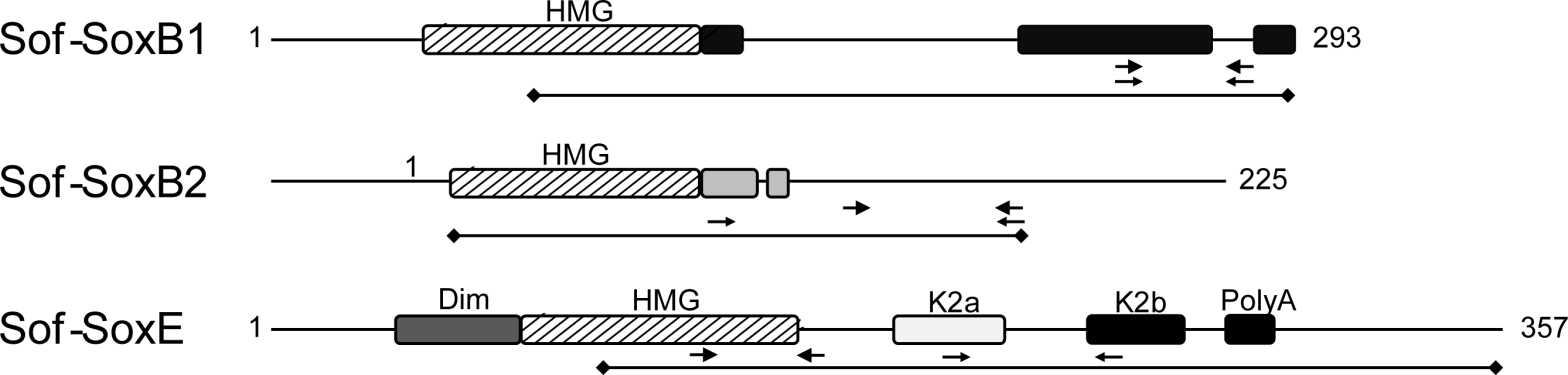
Predicted gene structure of cuttlefish Sox family genes isolated from *Sepia officinalis*. Regions of conservation with orthologous genes from other species are indicated by boxes (see text for details), including the high mobility group (HMG) for all three gene sequences. The Sof-SoxB2 sequence is incomplete at the 3’ end, indicated by the dashed line. Position of the primers used for RT-PCR analysis are indicated by small arrows, and for qPCR with large arrows. The portions of the gene sequence used for the in situ probes are also indicated (black line with diamonds).

Gene specific primers were designed based upon the HMG sequence for all three genes, and 3’- and 5’-RACE were used to extend the gene fragments in towards the C- and N-terminals respectively (Fig 2). We retrieved 1415 bp sequence for *Sof-SoxB1* (GenBank: KC545795) and 1143 bp for *Sof-SoxE* (GenBank: KC545796), including coding sequence and UTRs. RACE experiments for *Sof-SoxB2* resulted a 3-prime extension for a total of 676 bp (GenBank: KC545794). Alignment of retrieved coding sequences reveals conservation of known domains located outside of the HMG domain for each gene (Fig 2). A SoxB homology domain [47] is present in both *Sof-SoxB1* and *Sof-SoxB2*, as are gene-specific domains within the C-terminal. The *Sof-SoxE* predicted protein contains a conserved dimerization domain in the N-terminus (Dim: [48], [49]) and a context-dependent trans-activation domain (K2: [50], [51]) in the C-terminal. Of interest, the Sof-SoxE protein contains a poly-alanine domain that is absent from other SoxE orthologs, although poly-alanine domains are found within other classes of Sox proteins (in particular members of the SoxB-class: [43]).

### Temporal expression patterns

**Fig 3:**
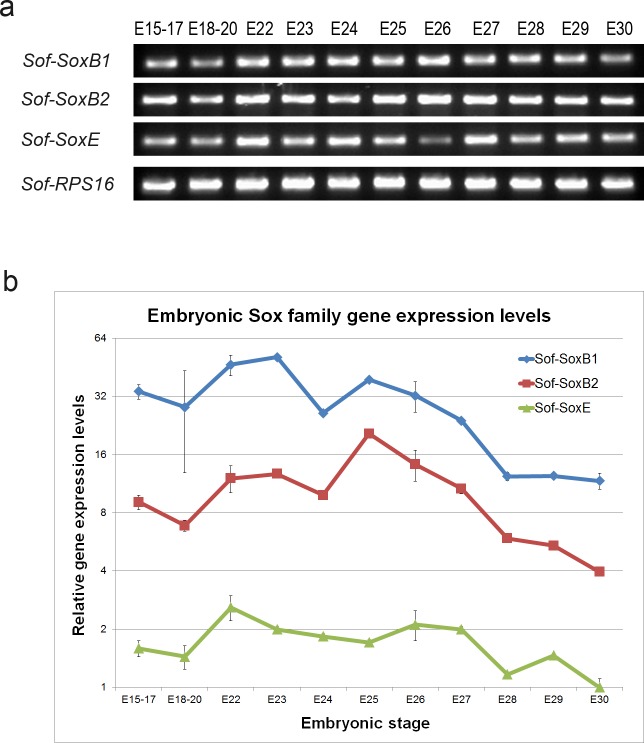
Temporal expression profile of *Sof-SoxB1*, *Sof-SoxB2* and *Sof-SoxE* during embryogenesis. Stage specific cDNA was used to amplify specific gene fragments in order to evaluate expression during development, using the ribosomal protein S16 (RPS16) as an internal control for template loading. a) RT-PCR: Amplification of gene fragments occurred for every stage tested, indicating gene expression throughout development. b) qPCR: Ct data for specific gene fragments were normalized to RPS16 levels as an endogenous control. Gene expression is shown as fold difference with respect to gene expression levels of *Sof-SoxE* at stage E30, plotted on a log2 scale for best visualization of the expression profiles. Both SoxB genes show similar expression profiles, although Sof-SoxB2 demonstrates a higher relative peak at stage E25 with respect to Sof-SoxB1. All three genes show reduction in expression levels to near E30 levels by stage E28.

Temporal expression of *Sof-SoxB1*, *Sof-SoxB2* and *Sof-SoxE* was examined with RT-PCR (Fig 3A) and qPCR (Fig 3B) using gene specific primers. Gene expression levels were lowest at stage E30 for all three genes, suggesting minimal expression post-embryogenesis. All three genes were detected in the earliest stages examined, and dropped to near E30 values by stage E28 –at which point all tissues have been formed and are undergoing histological differentiation [3]. Comparing expression levels relative to stage E30 *Sof-SoxE* expression illustrates relative transcript abundance of the three genes. *Sof-SoxB1*shows the highest levels of gene expression in whole embryo assays. The two SoxB genes show similar whole embryo expression profiles relative to pre-hatching levels (E30), however *Sof-SoxB2* shows a stronger peak in expression at stage E25.

### Spatial expression patterns

Cephalopod embryogenesis is traditionally separated into 30 embryonic stages [52], [53], [3]. Here we concentrate on the period of differentiation, from stages E24 through E27, although we comment briefly on SoxB1 and SoxE expression during the disc phase (stage E18: see below). Nomenclature of the developing nervous system follows that described in [54,55], [8], and [5]. For ease of comparison with other molluscan systems, we adopt the embryonic axes as described in [56] wherein the mouth and brachial arm crown corresponds to the ventral surface, and the funnel is located posteriorly.

#### Sof-SoxB1

**Fig 4:**
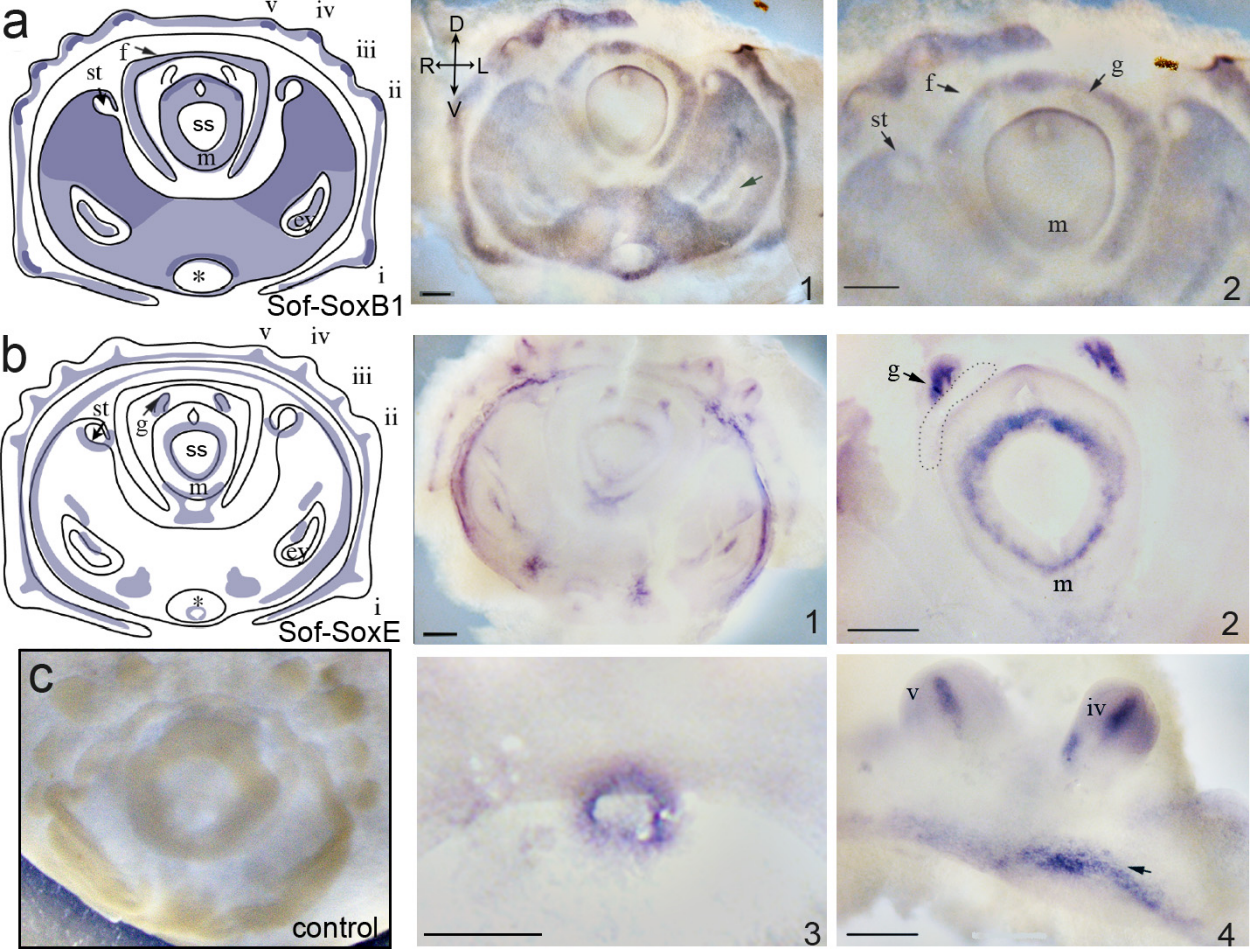
Whole mount *in situ* hybridization illustrating mRNA expression of Sof-SoxB1 and Sof-SoxE at stage E18 in the European cuttlefish *Sepia officinalis*. Column 1 shows a schematic illustration of stage-specific gene expression. **a) *Sof-SoxB1*** Expression is seen throughout the presumptive ectoderm, including expression within the developing eye (arrow); far right: higher magnification illustrating the absence of expression within the statocysts (st) and gill buds (g). **b) *Sof-SoxE*:** (1) Expression is found throughout presumptive mesoderm. (2) Higher magnification of a different embryo illustrating a ring of expression within the mesoderm underlying the forming mantle (m) and within the gill buds (g). The dash line indicates the area where hearts form in the pygmy squid [64] (3) A distinct ring of expression is also seen within the stomodeum. (4) Higher magnification illustrating medial expression within the arms and ring of underlying mesoderm (arrow). **c)** representative control embryo, sense probe. Abbreviations: *: stomodeum; ey: eye; f: funnel; g: gills; m: mantle; st: statocyst; i-v: arms. Scale bar in b3: 125μm; other scale bars: 500μm. body axis: D/V: dorsal/ventral. L/R: left/right.

**Fig 5:**
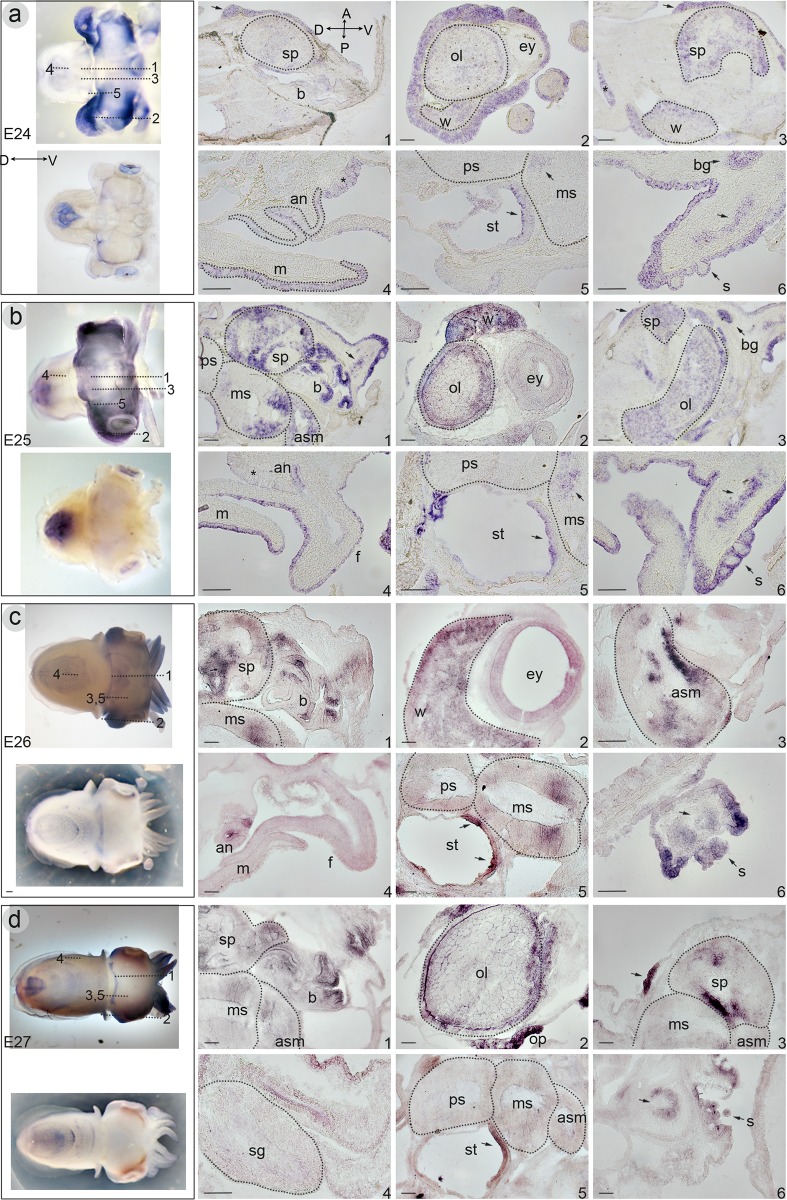
*In situ* hybridization illustrating mRNA expression of Sof-SoxB1 during embryogenesis of the European cuttlefish *Sepia officinalis*. Column on the left shows a representative wholemount stage-specific embryo indicating the position of each section represented in the panel (upper part) and a corresponding control embryo (lower part). Note expression in: **a) stage E24.** (1) the epithelium of the beak, in the presumptive nuchal organ (arrow) and in the supraesophageal mass. (2) the optic lobe and white bodies. (3) white bodies, supraesophageal mass, and in the presumptive nuchal organ (arrow). (4) epithelial layers of the mantle and the anus. (5) anterior statocyst epithelium (arrow), and also in the posterior portion of the medial subesophageal mass. (6) section through the arms; expression found surrounding the suckers, throughout the epithelium, and in the medial neuropil (arrow). The brachial ganglia at the base of the arms also shows expression. **b) stage E25.** (1) expression is retained in the beak, the supraesophageal mass, the posterior and anterior portion of the middle subesophageal mass, throughout the anterior subesophageal mass, and the epithelium and neuropil of the arms (arrow). (2) in the optic lobe and in the white bodies. (3) in the supraesophageal mass, optic lobe, brachial ganglia and in the epithelium, including the presumptive nuchal organ (arrow). (4) the mantle and funnel eptihelia. (5) expression as in stage E24. (6) section through the arms; expression as in stage E24. **c) stage E26.** (1) expression is retained in the beak, supraesophageal and middle subesophageal masses. Expression is no longer found within the epithelium overlying the head. (2) homogenous expression within the white bodies, (3) the anterior subesophageal mass shows concentration of expression around the neuropil, and reduced signal is evident ventrally. (4) expression is retained in the anal epithelium but is no longer found within the epithelium of the mantle or funnel. (5) the middle subesophageal mass and in the statocyst epithelium (arrows). (6) cross section of an arm; in signal is retained in the epithelium, suckers, and neuropil (arrow). **d) stage E27.** (1) expression is retained in the beak epithelium, and within the central nervous system as in previous stages. (2) expression is retained in the optic lobe and white bodies and within the epithelium of the optic pits. (3) expression is reduced within the middle and anterior subesophageal masses, and is concentrated around the neuropil of the supraesophageal mass. Expression is retained in the sensory epithelium of the nuchal organ (arrow). (4) higher magnification of stellate ganglia without Sof-SoxB1 localization. (5) signal is retained in the anterior statocyst epithelium (arrow), and very weak within the middle subesophageal mass. (6) section through the arms; expression is reduced within the in the epithelium, and is retained centrally within the sucker (s) and the neuropil (arrow). (*) in a4 and b4 indicates non-specific staining at the level of the funnel glands. Abbreviations: an:anus; asm: anterior subesophageal mass; b: beak; bg: brachial ganglia; ey: eye; f: funnel; g: gills; m: mantle; msm: middle subesophageal mass; spm: sopraesophageal mass; s: suckers; st: statocyst; psm: poster subesophageal mass; ol:optic lobes; op: optic pits; wb: white bodies; sg: stellate ganglion. body axis: A/P: anterior/posterior, D/V: dorsal/ventral. L/R: left/right Scale bars: 100μm.

*Sof-SoxB1* is expressed in all developmental stages examined. At the onset of organogenesis (stage E18) *Sof-SoxB1* is expressed within the overlying epithelium of the entire embryo (Fig 4A). Within the head, expression notably absent from the stomodeal opening, the invaginating statocysts, and the external region of the developing eyes. An expression domain is present in the center-most portion of the eyes. Expression is absent from the shell sac (ss) and the evaginating gill buds (g). From stages E24-E26 (left column, Fig 5), the level of expression within the mantle epithelium is reduced but expression in the head epithelium remains and is progressively restricted to the sensory epithelia of the nuchal organ (arrow; Fig 5D2) and olfactory pits (op; Fig 5D2).

Given the high level of epithelial expression within the head, which masks any potential underlying expression domains, we proceeded to analyse expression patterns within larger embryos (stages E24-E27) on frozen sections. No expression was found within the mantle cavity, including the stellate ganglia, at any stage investigated. At stage E24 expression is detected in the outer epithelial layer of the mantle, and within the epithelium of the anus (an; Fig 5A4) and the arms, including the suckers (s; Fig 5A6). Within the head, expression is seen within the epithelial layers of the developing beak (Fig 5A1), in the white bodies, and the lateral outer epithelial tissue (Fig 5A1 and 5A3). *Sof-SoxB1* expression is also found within lobes of the developing central nervous system (CNS) at this stage, specifically within the supraesophageal mass (Fig 5A3), in a few cells of the ventral-most portion of the middle subesophageal mass (arrows in Fig 5A5), in the ventral- most portion of the statocyst epithelium (Fig 5A5), within the brachial ganglia and the neuropil of the arms (arrows in Fig 5A6). By stage E25 expression is also evident in the outermost portion of the optic lobes (Fig 5B2) and throughout the anterior subesophageal mass (Fig 5B1). These expression domains remain stable through to stage E27 (Fig 5B–5D), with the exception of the signal within the mantle epithelium that is no longer detectable by E26 (Fig 5C4). At stage E27 expression within the subesophageal mass is reduced to nearly undetectable levels and becomes spatially restricted to the basal lobes of the supraesophageal mass (Fig 5D1–5D3), whereas within the optic lobes expression remains elevated in the outermost layers (Fig 5D2).

#### Sof-SoxB2

Expression of *Sof-SoxB2* within whole mount preparations is similar to the *Sof-SoxB1* gene, both genes showing expression within the outer epithelia of stage E24 embryos, masking expression domains from deeper within the embryo. Thus, we examine the spatial dynamics of *Sof-SoxB2* gene expression on serially sectioned embryos from stages E24-E27. Sequence alignment between the two cuttlefish SOXB genes confirms significant sequence divergence within the gene fragments used as *in situ* probes, thus we are confident that the gene expression patterns described for these two related genes accurately reflects gene specific expression and any potentially overlapping expression domains express both genes.

**Fig 6:**
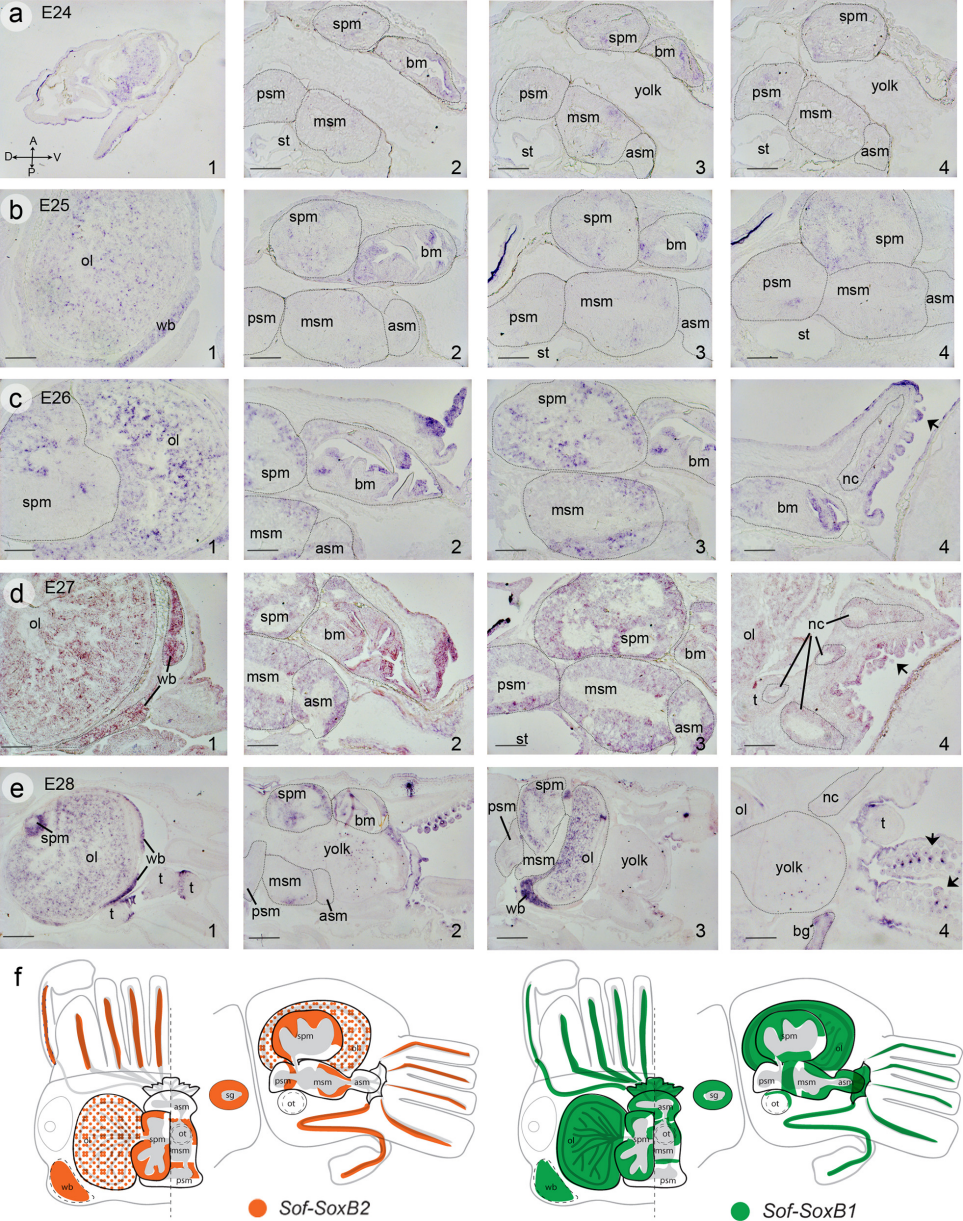
*In situ* hybridization illustrating mRNA expression of Sof-SoxB2 during embryogenesis of the European cuttlefish *Sepia officinalis*. **a) stage E24.** Lateral section through the entire embryo (1) and at the level of the head (2–4) showing expression with the in the buccal mass, cell in the anterior portion of the middle subesophageal mass, and cells near the neuropil of the posterior subesophageal mass. **b) stage E25.** A large homogeneously distributed population of cells are stained with in the optic lobes (1), while expression is retained within the buccal mass, and subesophgeal masses (2–4). Cells within the sopraesophageal mass also show specific staining at this stage (3, 4). **c) stage E26.** Expression is similar to stage E25, with staining retained in the optic lobes (1), sopraesophageal mass (1–3), middle subesophageal mass (2,3), and epithelia of the buccal mass (2, 4). Expression appears within the nerve cords of the arms at this stage, as well as within the overlaying epithelium of the arms (4). **d) stage E27.** Expression is similar to stage 26. **e) stage E28**. Expression begins to diminish within the lobes of the subesophageal mass (2), and appears within the epithelium of the tentacles, and the forming suckers of the arms. **f) schematic diagram** summarizing the expression domains of the two Sof-SoxB genes within the central nervous system: Sof-SoxB2 (orange) and Sof-SoxB1 (green). Abbreviations are as in Fig 5. Scale bars: 500μm.

At stage E24, similar to *Sof-SoxB1* gene expression, *Sof-SoxB2* expression is found within the epithelium covering the entire animal, including the head and brachial arms, and the entire mantle (Fig 6A1). Also similar to *SoxSoxB1* expression, *Sof-SoxB2* is detected within the epithelial layers of the developing beak within the buccal mass (Fig 6A2 and 6A3). At the level of the CNS, *Sof-SoxB2* expressing cell populations are detected with increasing intensity as development proceeds throughout the optic lobes and supraesophageal mass, with the exception of the central basal lobes where *Sof-SoxB1* is expressed. Similarly, within the subesophageal mass no expression is found within the anterior lobe, whereas *Sof-SoxB2* expressing cells are found within the ventral portion of the middle lobe in a location similar to the *Sof-SoxB1* expression domain, and in the cells surrounding the neuropil of the posterior lobe (Fig 6A4 and 6B4). By stage E25, expression within the epithelium of the anterior mantle and the head epithelium is no longer detected while it is retained posteriorly until stage E26. Expression appears within the brachial nerve cords of the arms at stage E26 (Fig 6C4) and remains through to stage E28 (Fig 6D4). Expression within the brachial epithelial of the arms and expression domains within the nervous system remain stable through to stage E28 (Fig 6E). A schematic diagram depicting potentially overlapping expression domains of the two SoxB gene is shown in Fig 6F; the most dramatic difference is the presence of *Sof-SoxB1* in the anterior supraesophageal mass, whereas *Sof-SoxB2* is absent. Within the optic lobes *Sof-SoxB2* appears to be restricted to a more homogeneous subpopulation of cells with respect to *Sof-SoxB1*.

#### Sof-SoxE

**Fig 7:**
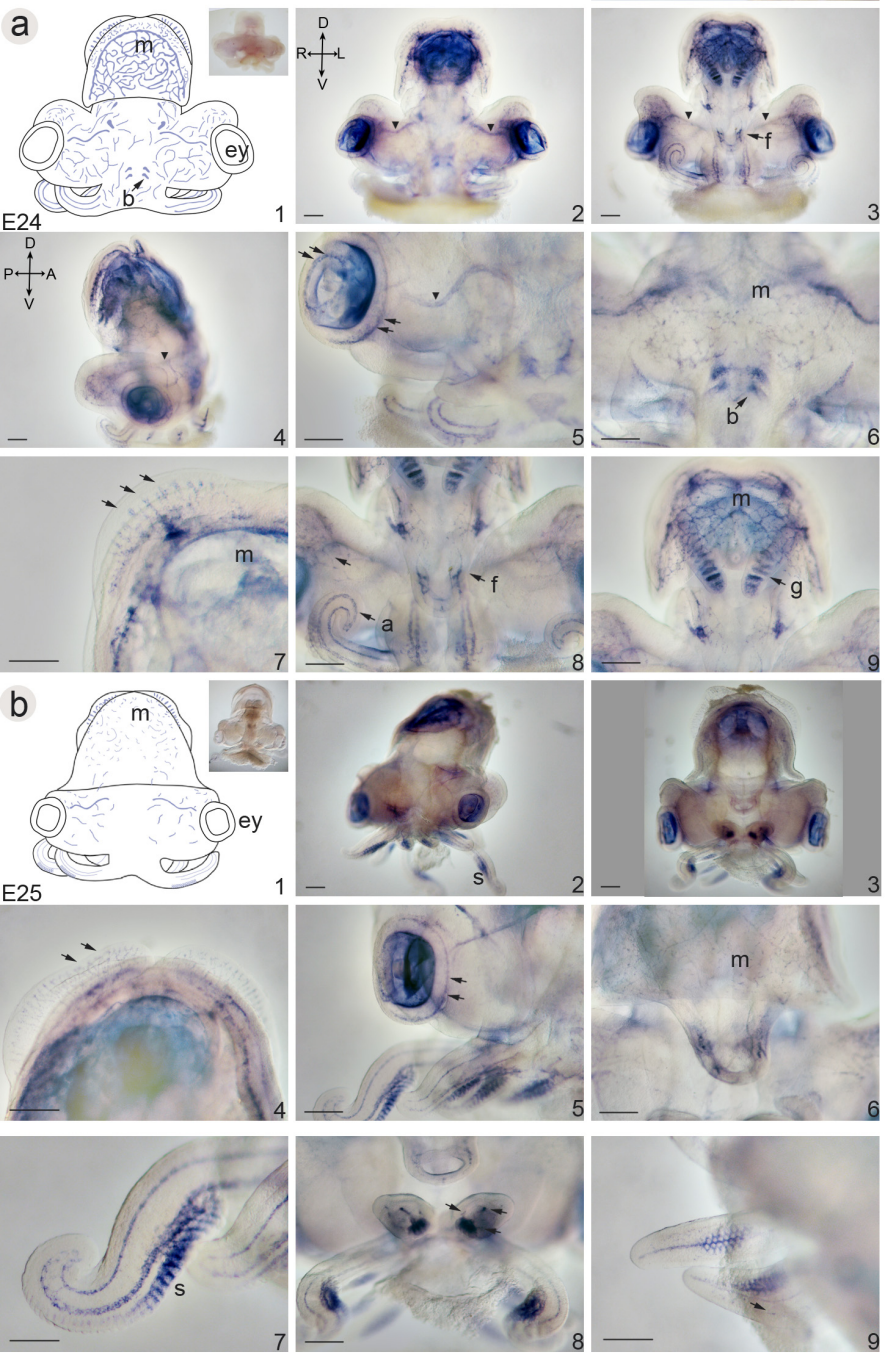
Whole mount *in situ* hybridization illustrating mRNA expression of Sof-SoxE during embryogenesis of the European cuttlefish *Sepia officinalis*. Column 1 shows a schematic illustration of stage-specific gene expression. Examples of control embryos (inset) show variation of non-specific colouration found within the eyes and developing cuttlebone. **a) stage E24**. (2) dorsal, (3) ventral and (4) lateral views of a stage E24 embryo showing extensive expression at the level of the developing circulatory system. The ophthalmic vessels are particularly evident (arrowheads); (5–9) higher magnification to illustrate details. (5) anterior right side of the head showing the ophthalmic vessels (arrowhead) and specific expression within the eyes (arrows). (6) Anterior-medial view of the head showing expression at the level of the developing beak (b). Notice also the fine capillary network of the head in this region. (7) Anterior view of developing fins highlighting the expression with the network of vessels (arrows). (8) Posterior view at the level of the funnel. Expression within the vasculature of the funnel (f), arms (a) and capillaries of the head (arrow) is evident. (9) Posterior view of the mantle (m) showing the intricate network of vessels, and signal within the gills (g). **b) stage E25**. (2) anterior and (3) posterior views illustrating a reduction of expression at the level of the circulatory system. (4–9) higher magnification to illustrate details. Lower levels of expression with respect to stage E24 are retained within the circulatory system of the fins (4), within the eyes (5), and within the mantle (6). (7–9) higher magnification illustrating expression details within the arms and tentacles. (7) Expression is evident both anteriorly and posteriororly within the tentacles, as well as within the ventral most suckers (s) on the posterior side. (8) An optical cross section though the arms at this stage reveals expression corresponding to the three posterior vessels (arrows) in addition to the sucker staining. (9) Posterior view of the expression within the suckers illustrates strong signal within the posterior vessel and tissues surrounding the developing suckers, as well as the formation of the lateral blood vessels (arrow). Abbreviations are as in Fig 5. Scale bars: 500μm.

Wholemount *in situ* hybridization demonstrates that at stage E18 *Sof-SoxE* is expressed in the presumptive mesodermal territories (Fig 4B). Expression is found throughout the base of arm territory, and is concentrated medially within the brachial arm buds (arms i-v in Fig 4). A distinct expression domain surrounds the entire head-region, separating the head from the arm territory. Signal is also evident within cells underlying the developing mantle, in the gill buds (Fig 4), and in isolated paired patches of cells near the statocysts, eyes, and stomodeum. A distinct ring of cells is found within the stomodeum. Expression domains change once the embryo has passed into the straightening phase. At stage E24 expression is visible as a net of interconnected cells which correspond to the developing vascular system on anterior (Fig 7A2), posterior (Fig 7A3) and lateral surfaces (Fig 7A4): vessels are present on either sides of the mantle muscles (Fig 7A7 and 7A9), in the developing gills (Fig 7A9), in the fin (Fig 7A7, arrows) and in the funnel tissues (Fig 7A8). The developing vasculature within the head also expresses *Sof-SoxE*, with the ophthalmic vessels particularly evident (Fig 7A2, 7A3, 7A4 and 7A5, arrowhead). Cells within the developing eyes (Fig 7A5) and in the beak (Fig 7A6) also show expression; within the developing arms expression remains underneath the epithelium within two distinct rows of cells (Fig 7A8). Expression within the vascular network begins to diminish at stage E25 (Fig 7B2 and 7D3), except for in the arms where expression remains strong within the developing vessels and signal is also found within the developing suckers (s: Fig 7B7–7B9, arrows). By late-stage E26, expression within the superficial vasculature has receded, and any additional expression domains appear restricted to deep layers of the developing embryo which are difficult to identify in whole mount specimens.

**Fig 8:**
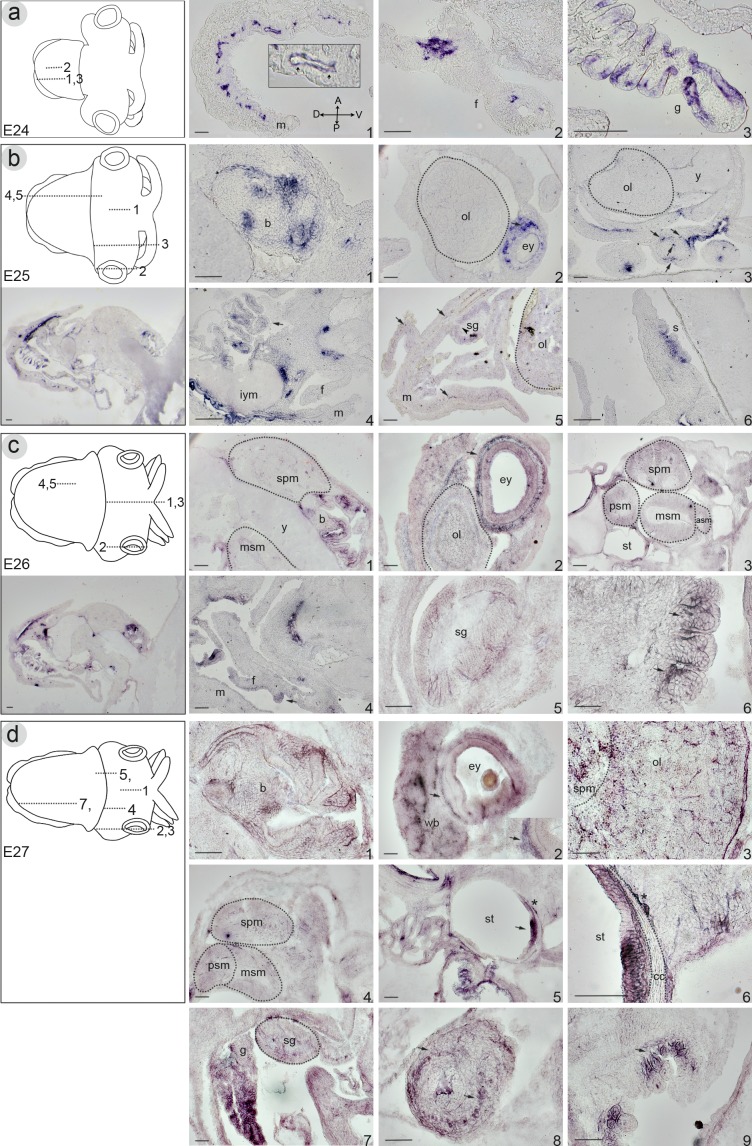
*In situ* hybridization on frozen sections illustrating mRNA expression of Sof-SoxE during embryogenesis of the European cuttlefish *Sepia officinalis*. Column on the left shows a schematic illustration of stage-specific embryo indicating the position of each section represented in the panel (upper part) and a medial section showing the whole embryo and its orientation (lower part). Note expression in: **a) stage E24**. (1 the vascular network of the mantle. The tubular structure is particularly evident (inset: small arrows). (2) extensive staining at the base of the funnel. (3) the gill epithelium. **b) stage E25.** (1) the mesoderm of the developing beak. (2) within the connective tissues surrounding the eye and absent from the optic lobe. (3) in four main vessels of the arms (arrows). (4) signal is evident in the mesenchyme. (5) expression is retained in vascular system of the mantle and funnel tissues (arrows) and appears in the stellate ganglia. (6) section through the arms; expression is localized in the mesenchyme under the epithelium of the developing suckers. **c) stage E26**. (1) expression is conserved in the mesenchyme of the beak and appears in scattered cells of the supraesophageal and middle subesophageal masses. (2) signal is retained in the connective tissues surrounding the eye and appears in the optic lobe. (3) weak expression localized in the supraesophageal mass and all three lobes of subesophageal masses, and the dorsal-anterior portion of the statocyst epithelium. (4) expression continues to be present in the mesenchyme of various organ in the mantle cavity. (5) expression is retained in the stellate ganglia. (6) section through the arms; expression as in stage E26 at the base of the suckers (arrows). **d) stage E27.** (1) the mesenchyme of the beak. (2) the connective tissues surrounding the eyes (arrow, inset) and appears in the white bodies. (3) cells of the optic lobe and supraesophageal mass. (4) weak expression within cells of the middle subesophageal mass and supraesophageal mass. (5) the statocyst epithelium (6) higher magnification of at the level of the statocyst; Cells lining the statocyst cartilage show high levels of expression, whereas the chondrocytes do not express *Sof-SoxE*. (7) lateral section of the mantle cavity; expression is retained in the developing gills and in the stellate ganglia. (8) Section through the tentacles; expression is found within the neuropil and connective tissues. (9) Section through the arms; expression is retained within the mesenchyme underlying the suckers. Scale bars: 100μm. Abbreviations: cc: cartilage condensations; all others as in Fig 5.

At stage E24, sectioned material confirms *Sof-SoxE* expression in mantle vasculature (Fig 8A1), in the funnel tissues (Fig 8A2) and in the developing gills (Fig 8A3). At stage E25, within the developing beak *Sof-SoxE* shows high levels of expression within the mesodermal tissues underlying the *Sof-SoxB1/2* positive epithelium (Fig 8B1). No expression is seen within the various lobes of the developing nervous system at this time whereas strong signal is visible within the connective tissues surrounding the eye (Fig 8B2). In addition to the developing vascular system within the differentiating mantle and funnel tissues (Fig 8B5, arrows), *Sof-SoxE* expression is associated with the four main vessels of the arms (Fig 8B3, arrows). Expression is evident in the mesenchyme underlying various epithelial organs within mantle cavity (Fig 8B4) and in the stellate ganglia (sg: Fig 8B5). Whereas *Sof-SoxB1* expression is found within the epithelial layers of the developing suckers, *Sof-SoxE* is expressed within the underlying mesenchyme (Fig 8B6). Mesenchymal expression domains remain through stage E27, whereas expression within the vascular system is no longer detectable by stage E26. At this stage expression also appears within the central nervous system, including the supraesophageal mass (Fig 8C1), the optic lobes (Fig 8C2), as well as within cells within the anterior, middle and posterior subesophageal masses (Fig 8C3). Signal is retained within the stellate ganglia, and elevated expression within the ventral portion of the statocyst epithelium is observed (Fig 8C3). Expression appears within cells of the white bodies at stage E27 (Fig 8D2) and is expanded within the central nervous system, showing elevated cell numbers within the optic lobes (Fig 8D3) and cells within the connecting neuropil that joins the optic lobes with the more posterior supraesophageal mass (Fig 8D4). Expression is evident in cells lining the statocyst cartilage (Fig 8D5 and 8D6); the chondrocytes themselves are not labelled by our probe. In fact, although mesenchymal cells throughout the embryo express *Sof-SoxE* in all stages examined, expression was not found within cartilage condensations at any time point in this study. By stage E28, expression is retained in some cells within the stellate ganglia (Fig 8D7 and 8D8), throughout the gills (Fig 8D7), and at the base of the suckers (Fig 8D9 and 8D10), and an additional expression domain appears within the neuropil of the tentacles.

## Discussion

We report on the cloning and expression of two Sox gene families (SoxB and SoxE) from the European cuttlefish, *Sepia officinalis*. This is the first report of Sox family genes from cephalopod molluscs, and contributes to the relatively sparse knowledge of this gene family from lophotrochozoan lineages in general.

### Sof-SoxB

Buresi [6] *et al* have recently characterized the development of sensory epithelium and peripheral nervous system in the European cuttlefish, through the expression of the *Sof-pax3/7* and *Sof-elav1* genes. We show here that while *Sof-SoxB1* is initially expressed more broadly in the ectoderm with respect to *Sof-pax3/7*, which expands to encompass the entire outer epithelium [6], *Sof-SoxB1* expression is progressively restricted to sensory epithelium as development proceeds (the olfactory pits, the statocysts, the suckers, and the nuchal organ). LeGuoar *et al* [19] report on the expression of SoxB1 in the marine gastropod *Patella vulgata*, demonstrating expression in cells that give rise the sensory apical organ as well as additional ectodermal domains that the authors state likely correspond to regions that produce neural structures in the adult. Romagny *et al* [57] have reported data indicating that embryos as early as stage E23 are responsive to both tactile and chemical cues from within the embryonic egg capsule. In vertebrates, SoxB1 members (Sox 2 and 3) are expressed in sensory cell progenitors, in particular within the sensory epithelium of the inner ear (chick: [58]; mouse: [59]) and olfactory epithelia [60]. Taken together, these data lead to the speculation that Sof-SoxB1 may play a similar role regulating sensory cell differentiation within the epithelium of the developing cuttlefish.

In general, SoxB1 family genes are known as transcriptional activators whereas the SoxB2 family functions as repressors [47]. In vertebrates, the SoxB2 gene Sox21 has been shown to repress Sox3 (SoxB1) expression and promote terminal neural differentiation during embryogenesis [61], as well as inducing stem cell differentiation [62]. To date few data are available regarding expression patterns of both SoxB family members from invertebrate lineages, however where investigated overlapping expression domains have been reported. The high degree of potentially overlapping SoxB1/B2 expression domains described here conform to this general pattern. Interestingly, areas of differential expression are evident. Within the optic lobes, *Sof-SoxB2* is expressed in a larger cell population with respect to *Sof-SoxB1*, which is restricted to the outermost layers. Within the subesophageal mass, *Sof-SoxB1* appears to be more expansive in the anterior and middle lobes with respect to *Sof-SoxB2*. Potentially overlapping expression domains are present within the middle subesophageal mass, however *Sof-SoxB2* is expressed in the posterior subesophageal lobe where *Sof-SoxB1* expression is absent. These data point to distinct functional roles for the two SoxB genes within the developing CNS of the cuttlefish.

### Sof-SoxE

We demonstrate that *Sof-SoxE* is expressed throughout the presumptive mesoderm at the onset of organogenesis. One mesodermal tissue of particular interest with in the cephalopod lineage is their vertebrate-like cartilage [1], [2] [3]. A vertebrate SoxE gene, Sox9, is fundamental for vertebrate cartilage formation, thus we were particularly interested in the potential role of *Sof-SoxE* within the developing cuttlefish cartilages. *Sof-SoxE* shows expression in undefined connective tissues within the mantle, however we find no evidence of expression in the pre-cartilage cell condensations [3]. It is of interest to note that connective tissue cells found at the periphery of cartilages that form through condensations (such as the cranial cartilage in Fig 8D6) show high levels of Sof-SoxE expression. Thus we cannot rule out the possibility of migratory Sof-SoxE expressing connective tissue cells contributing to cartilage formation, especially in mantle-derived cartilages where no pre-cartilage condensations form (see [3]).

While *Sof-SoxE* does not appear to be involved in chondrogenesis, it is involved in vasculargenesis of the circulatory system; *Sof-SoxE* is expressed in the cells that form the architecture of the developing peripheral circulatory system throughout the body at the on-set of vessel formation. We infer that these are blood vessels based upon these criteria: the main vessel tracks in the head correspond to locations of major blood vessels well described in adult cuttlefish [63], for example the ophthalmic vessel of the head is well delineated in our samples (see arrowheads in Fig 7A). The histology of these structures as hollow vessels is also apparent (see inset in Fig 8A1). Moreover, in later material the same tubular structures that are no longer Sof-SoxE positive have the histological properties of haemolymph (data not shown); they show light pink to blue staining from the colouration protocol used, a property that is shared with the yolk sac and the blood sinus cavities of the head (see iym in Fig 8B).

We also find Sof-SoxE expression within connective tissues surrounding the abdominal sinus cavities at the earliest stage examined in section (iym: Fig 8B4). Studies on other cephalopods indicate that the hearts are thought to derive from these connective tissues. For example, Yoshida *et al*. [64] report on the formation of the circulatory system in the pygmy squid *Idiosepius*, wherein they demonstrate that the vascular tubes which form the heart are present as early as stage E21. We do not examine embryos in histological section prior to stage E24, however early Sof-SoxE expression within the mesoderm at stages E18-E20 may indeed include the heart cell precursors. No expression is seen in areas that roughly correspond to the location of heart precursors in the pygmy squid (dashed line in Fig 4B2), however a detailed study of heart formation in *S*. *officinalis* is not currently available. Expression in the circulatory system is a novel expression domain for a SoxE- family gene. We note however, the close phylogenetic relationship between the SoxE and SoxF family members (Fig 1). Members of the SoxF family (Sox7, Sox 17, and Sox18) have been identified within vertebrate systems as being important for vessel formation (reviewed in [65]. SoxF expression within invertebrate lineages is variable; within Drosophila, SoxF is expressed in the peripheral nervous system [66], whereas in the honeybee the SoxF homolog shows ubiquitous embryonic expression [25]. The nematode *C*. *elegans* lacks a SoxF gene [43] and SoxF homologs in cnidarians and ctenophores show endodermal expression (reviewed in [67]). We were unable to identify a SoxF homolog with our search strategy and thus comment upon the presence or absence of a bona fide SoxF homolog awaits genome sequencing of this organism.

Sof-SoxE is also expressed within the developing nervous system, indicating a transition from predominantly connective tissue early expression, to playing a putative role in the function and maintenance of the nervous system. We find *Sof-SoxE* expression all lobes of the CNS, as well as within the peripheral stellate ganglia. The expression domain within the various lobes of the brain expands as development proceeds. From our data, we can speculate that the *Sof-SoxE* gene has been incorporated into the generation of two novel cephalopod features: the closed peripheral circulatory system and the sophisticated centralized nervous system.

## Conclusions

Slowly, the genetic machinery underlying the generation of the complex cephalopod anatomy is being revealed. Most published studies to date have focused on the development of the central nervous system [5], [8], [7], [9], [10], [11], [12], [13]. Here we contribute data that expands our knowledge of the genetic control of two other cephalopod novelties: the closed peripheral circulatory system and the cellular cartilages. We demonstrate the absence of SoxE expression within early cartilage cell condensations, and show instead that this gene is involved in the formation of the circulatory system. In addition, we show that three sox family members, *Sof-SoxB1*, *Sof-SoxB2* and *Sof-SoxE* are expressed within the developing CNS, thus adding to the repertoire of genes known to play a role in cephalopod nervous system development.
